# Preclinical characterization of the novel anti-SIRPα antibody BR105 that targets the myeloid immune checkpoint

**DOI:** 10.1136/jitc-2021-004054

**Published:** 2022-03-07

**Authors:** Zhen-Hua Wu, Na Li, Xiao-Feng Mei, Juan Chen, Xiao-Ze Wang, Ting-Ting Guo, Gang Chen, Lei Nie, Yao Chen, Mei-Zhu Jiang, Ji-Teng Wang, Hai-Bin Wang

**Affiliations:** 1 BioRay Pharmaceutical Co., Ltd, Taizhou, Zhejiang, China; 2 BioRay Pharmaceutical Corp, San Diego, California, USA

**Keywords:** immunotherapy, drug evaluation, preclinical, immunity

## Abstract

**Background:**

The CD47-SIRPα pathway acts as an important myeloid cell immune checkpoint and targeting the CD47/SIRPα axis represents a promising strategy to promote antitumor immunity. Several CD47-targeting agents show encouraging early activity in clinical trials. However, due to ubiquitous expression of CD47, the antigen sink and hematologic toxicity, such as anemia and thrombocytopenia, are main problems for developing CD47-targeting therapies. Considering the limited expression of SIRPα, targeting SIRPα is an alternative approach to block the CD47-SIRPα pathway, which may result in differential efficacy and safety profiles.

**Methods:**

SIRPα-targeting antibody BR105 was generated by hybridoma fusion and following humanization. BR105 was characterized for binding to human SIRPα alleles and blockade of the interaction with CD47. The functional activity was determined in in vitro phagocytosis assays by using human macrophages. The effect of BR105 on human T cell activation was studied using an OKT3-induced T-cell proliferation assay and an allogeneic mixed lymphocyte reaction. Human SIRPα-humanized immunodeficient mice were used in cancer models for evaluating the in vivo antitumor efficacy of BR105. Safety was addressed in a repeat-dose toxicity study in cynomolgus monkeys, and toxicokinetic analysis was further evaluated.

**Results:**

BR105 shows broad binding activity across various SIRPα variants, and potently blocks the interaction of SIRPα and CD47. In vitro functional assays demonstrated that BR105 synergizes with therapeutic antibodies to promote phagocytosis of tumor cells. Moreover, the combination of BR105 and therapeutic antibody significantly inhibits tumor growth in a xenograft tumor model. Although BR105 may slightly bind to SIRPγ, it does not inhibit T cell activation, unlike other non-selective SIRPα-targeting antibody and CD47-targeting agents. Toxicity studies in non-human primates show that BR105 is well tolerated with no treatment-related adverse effects noted.

**Conclusions:**

The novel and differentiated SIRPα-targeting antibody, BR105, was discovered and displays promising antitumor efficacy in vitro and in vivo. BR105 has a favorable safety profile and shows no adverse effects on T cell functionality. These data support further clinical development of BR105, especially as a therapeutic agent to enhance efficacy when used in combination with tumor-targeting antibodies or antibodies that target other immune checkpoints.

## Introduction

Cancer cells escape from immune surveillance by several mechanisms, such as recruitment of immunosuppressive cells, lost tumor antigenicity, and enhanced expression of inhibitory immune checkpoints in the tumor microenvironment.^1^ Relieving immune suppression is an important strategy for improvement of antitumor immunity and the development of anticancer drugs. Antibodies against immune checkpoints programmed cell death 1 (PD-1), programmed death ligand 1 (PD-L1), or cytotoxic T-lymphocyte antigen-4 (CTLA-4), are breakthroughs in cancer immunotherapy. These checkpoint inhibitors significantly improve survival outcomes for patients with metastatic cancer.^2–4^ However, it was reported that many patients either did not respond to checkpoint blockade or developed acquired resistance, which led to cancer progression or relapse.^5–8^ Targeting alternative checkpoints or combination of immune checkpoint inhibitors may be effective ways to improve antitumor immune response.

In contrast to PD-1/PD-L1 and CTLA-4, which are well-established T-cell immune checkpoints and target these checkpoints would enhance adaptive immune, the CD47/SIRPα is a myeloid-specific immune checkpoint for innate immune cells.^9–11^ The SIRPα–CD47 interaction transmits a ‘do not eat me’ signal to macrophages and other myeloid cells. CD47 is broadly expressed in normal cells and serves as a marker of ‘self’ to prevent phagocytosis of healthy cells.^12 13^

It has been reported that CD47 is overexpressed in numerous human cancers to escape phagocytosis, and increased CD47 expression is correlated with poor prognosis in various hematologic and solid tumors.^14 15^ The CD47/SIRPα axis is emerging as a promising target in cancer therapy. Antibodies targeting the CD47/SIRPα axis induce enhanced macrophage phagocytosis of tumor cells.^14 16 17^ Moreover, targeting the CD47/SIRPα axis suppresses tumor growth in vivo, or synergizes with tumor-targeting monoclonal antibodies, as well as immune checkpoint inhibitors.^18–21^ Numerous CD47/SIRPα blocking agents have been explored and are currently being evaluated in clinical trials, including anti-CD47 antibodies, anti-SIRPα antibodies and SIRPα-Fc fusion proteins. However, due to the broad expression of CD47, treatment with CD47-targeting agents have caused significant anemia and thrombocytopenia in both preclinical studies and clinical trials,^22–24^ and higher therapeutic doses of CD47-targeting agents are needed to overcome the antigen sink and block CD47. In addition, CD47 could also interact with other proteins, such as integrins, thrombospondin-1 and SIRPγ. The CD47 signaling appears to have a more complex biological function and CD47 block may elicit unexpected cellular responses.^25 26^

Due to the more limited tissue expression pattern of SIRPα, targeting SIRPα may be a promising way to block the CD47/SIRPα axis. SIRPα belongs to the paired receptor family of closely related SIRP proteins. SIRPα is expressed on many myeloid cells, including monocytes, macrophages, dendritic cells, granulocytes, and neurons.^27^ Binding of CD47 to SIRPα induces phosphorylation of the intracellular immunoreceptor tyrosine-based inhibitory motifs (ITIMs) and activates the inhibitory phosphatases SHP-1 and SHP-2.^9 28 29^ This cascade ultimately suppresses the function of non-muscle myosin IIA and restricts phagocytic function.^30^

In the current study, we report the discovery and development of a novel anti-SIRPα antibody, BR105, as an approach to deliver the therapeutic benefit of SIRPα-CD47 blockade. BR105 recognizes the most common allelic SIRPα variants in humans. Binding of BR105 to SIRPα blocks its interaction with CD47, thereby promoting macrophage phagocytosis of cancer cells. Our studies demonstrate that BR105 synergizes with other therapeutic antibodies to promote phagocytosis of tumor cells and inhibits tumor growth in a xenograft tumor model. Although BR105 binds to SIRPγ slightly, it shows no adverse effects on T cell functionality, while other non-selective SIRPα-targeting antibody and CD47-targeting agents impair human T cell activation and proliferation. BR105 is a novel, differentiated SIRPα-targeting antibody that shows promising efficacy and a favorable safety profile in vitro and in vivo. These data support the future development of BR105 in combination with other anticancer drugs.

## Methods

### Antibody development and humanization

Anti-human SIRPα antibodies were developed by standard hybridoma technology. Hybridomas were selected, and supernatants from the resulting clones were screened by SIRPα binding and blocking assays. One of the hybridoma clones, termed 7C2, was cloned and sequenced. Humanization of 7C2 was performed by CDR-grafting onto human germline frameworks. The humanization version of 7C2 was designated as BR105.

### mAb generation

Expression vector encoding antibody heavy chain and light chain of BR105 was transfected into CHO-S cells using freestyle MAX (Gibco), and the monoclonal stable cell line was generated by MTX selection and limiting dilution. For BR105 production, cells were cultured in 300 L bioreactors for 14 days in a fed-batch mode, and BR105 was purified using protein A affinity chromatography and ion exchange chromatography. 18D5,^31^ KWAR23,^32^ 1H9^33^ and SIRP4^34^ on a human IgG1 backbone with N297A amino acid substitution in the Fc region backbone were expressed by Biointron Biological. Human IgG1 control, human IgG4 control, mouse IgG1 control and Hu5F9 were purchased from Biointron Biological. B6H12 was from BioXcell. Anti-human CD20 antibody (zuberitamab) and anti-HER2 antibody (Herceptin biosimilar, HS022) were produced in-house.

### Cell lines and cell culture

Human tumor cell lines U937, THP-1, Raji and SK-BR-3 were obtained from the American Type Culture Collection and cultured as recommended by the vendor. Human PBMCs, CD14+ monocytes, neutrophils and dendritic cells were purchased from AllCells.

### Binding to SIRPα and SIRPγ by ELISA

Human SIRPα V1, SIRPα V2, SIRPα V8 (ACROBiosystems), SIRPγ (Sino Biological) or cynomolgus monkey SIRPα (ACROBiosystems) were coated on 96-Well ELISA plates (Corning) at 0.5 µg/mL in PBS, blocked with 5% BSA in PBS, and incubated with serially diluted indicated mAbs. The samples were then incubated with HRP-labeled Goat anti-human IgG Fc secondary Ab (Sigma). Peroxidase substrate TMB (InnoReagents) color development was stopped by adding 1 M H_2_SO_4_ and absorbance was read at 450 nm on a microplate reader (Molecular Devices). A dose response curve was fitted by 4‐parameter logistic (4PL) regression (Prism V.8; GraphPad).

### Antibody affinity measurement

Affinity experiments were performed on an Octet RED96 (ForteBio) at 25°C. The test Abs were captured onto anti-human IgG Fc capture (AHC) biosensors (ForteBio). Measurements were made with serial dilutions of human SIRPα-His fusion proteins (Sino Biological). The association of the antigen was measured for 40 s, followed by a dissociation step for 100 s. Curve fitting was performed using a 1:1 binding model and the ForteBio data analysis software V.9.0 (ForteBio).

### Cell-based SIRPα binding

SIRPα binding was studied using THP-1 and U937 AML cell lines. The cells were incubated for 0.5 h at 4°C with increasing concentrations of BR105. The cells were then stained with FITC-labeled goat anti-human IgG conjugates (Abcam), and analyzed by flow cytometry (Calibur, BD Biosciences).

For binding to CD14+ monocytes, macrophages and neutrophils, cells were blocked with human Fc receptor Blocking Reagent (BD Biosciences) for 20 min at room temperature. The cells were then incubated for 0.5 h at 4°C with 10 µg/mL BR105. Bound antibodies were detected using Goat anti-Human IgG F(ab')_2_-FITC conjugates (Invitrogen), and cells were analyzed by flow cytometry (Calibur, BD Biosciences).

### Blocking CD47 binding to SIRPα by ELISA

The 96-well plates (Corning) were coated with Fc-tagged CD47 (ACROBiosystems) in PBS at 4°C overnight. The plates were blocked with 5% BSA in PBST for 1 h at 37°C. Serially diluted BR105 and biotinylated SIRPα V1, SIRPα V2, SIRPα V8 (ACROBiosystems) were added to the plates. After incubation at 37°C for 1 h, bound SIRPα was detected by adding streptavidin-HRP (Abcam). Then the plates were washed and the substrate TMB (InnoReagents) was added. The reaction was stopped by adding 1 M H_2_SO_4_. Absorbance was read at 450 nm on a microplate reader (Molecular Devices).

### Blocking human CD47 binding on SIRPα-expressing cells

U937 cells or macrophages were blocked with human Fc receptor Blocking Reagent (BD Biosciences) for 20 min at room temperature. Then cells were incubated with 10 µg/mL of murine IgG2a Fc-tagged human CD47 (ACROBiosystems) in the absence or presence of BR105. Binding of CD47 on the cells was measured by adding Goat anti-mouse-IgG-APC (R&D Systems) and analyzed by flow cytometry. Binding was normalized to the mean fluorescence intensity of CD47-SIRPα binding in the absence of BR105.

### Phagocytosis assay

The PBMCs (AllCells) were washed once in PBS and resuspended in RPMI-1640. The cells were then plated in a culture plate and allowed to adhere in a 5% CO_2_ container at 37°C for 2 h. Non‐adherent cells were removed by thorough washing with RPMI‐1640.

The adherent monocytes were cultured in medium (RPMI‐1640 (Gibco), 10% fetal bovine serum (Gibco)) containing 80 ng/mL human monocyte colony stimulating factor (Sino Biological) for 7 days at 37°C in 5% CO_2_ in order to obtain macrophages. Raji or SK-BR-3 were labeled with CFSE (Abcam) at 37°C for 10 min, and added to the macrophages (at a tumor cells:macrophages ratio of 2:1). BR105 alone, tumor-targeting mAb alone, or a combination of the two antibodies were added and incubated for 2 h at 37°C. At the end of incubation, the cells were collected and incubated with allophycocyanin-labeled anti-human CD14 (invitrogen) for 30 min. Phagocytosis was determined by flow cytometer and defined as the percentage of CFSE+ cells within the CD14+ population.

### OKT3-induced T-cell proliferation

96-well plates were coated with 0.1 µg/mL OKT3 (ACROBiosystems) at 4°C overnight. Then human PBMCs were seeded in OKT3-coated plates, treated with 10 µg/mL of indicated mAbs, and incubated for 3 days at 37°C in 5% CO_2_. Cell proliferation was determined using the CellTiter-Glo kit (Promega).

### Allogeneic mixed lymphocyte reaction

Dendritic cells (AllCells) were treated with 50 µg/mL Mitomycin C (Selleck) at 37°C for 30 min and then plated onto a 96-well plate at 5×10^3^ cells per well. Allogeneic PBMCs (AllCells) from different donors were added at a dendritic cell:PBMCs ratio of 1:20. Indicated mAbs were added at a concentration of 10 µg/mL immediately, and the cells were incubated at 37°C in 5% CO_2_ for 5 days. Interferon (IFN)γ secretion were evaluated in the supernatant by ELISA (R&D Systems).

### Mouse tumor xenograft model

For tumor cell engraftment, 1×10^5^ Raji-Luciferase were injected intravenously into SIRPα-humanized B-NDG mice (Biocytogen, Beijing, China). Bioluminescent imaging was performed to monitor tumor growth. Treatment was initiated 3 days post-engraftment when total flux reached approximately 1.25×10^6^ p/s (n=8 for each group). Mice were injected with human IgG1, anti-human CD20 antibody (zuberitamab, 0.1 mg/kg), BR105 (10 mg/kg), or a combination of anti-human CD20 and BR105. Anti-human CD20 was administered via intravenous injection on day 0 and 10 of post treatment initiation, and BR105 was administered via intraperitoneal injection two times per week for a total of six doses. Total flux measurements were obtained two times a week to assess tumor growth.

### Toxicity study in non-human primates

The non-human primate toxicity study in cynomolgus monkeys was performed at Joinn Laboratories (Taicang, China) and was conducted in accordance with the US Food and Drug Administration Good Laboratory Practice (GLP) Regulations, 21 Code of Federal Regulations Part 58. The study plan was reviewed and approved by the Institutional Animal Care and Use Committee in compliance with the Guide for the Care and Use of Laboratory Animals (eighth Edition, The National Academy Press, Washington, DC) and other animal welfare standard (Public Law 99–198, US Department of Agriculture). Animals (five males and five females per group) were administered intravenous infusion of BR105 (15, 50 or 150 mg/kg) or vehicle control (10 mM L-Histidine pH 6.0) one time per week for 4 weeks (total of five doses). Following the dosing period, two animals/sex per group were maintained for a 6-week recovery period. In-life evaluations included clinical observations, body weight, food consumption, cardiovascular safety pharmacology evaluations, ophthalmologic examinations, clinical pathology (serum chemistry, hematology, coagulation and urine analysis). Gross pathology, relative organ weight and histopathological examination were performed. For toxicokinetic analysis, blood samples were collected pre-dose and at 0.25, 0.5, 2, 4, 8, 24, 48, 72, 96 and 168 h after initiation of the infusion on Days 1 and 22. In addition, blood samples were taken at 0.5 h after initiation of the infusion on Day 8, Day 15 and Day 29, and pre-dose on Day 15. The concentration of BR105 in the serum samples was determined by using a qualified immunoassay. A non-compartmental module of WinNonlin (8.0.0.3176) was used to calculate toxicokinetic parameters.

### Statistics

Data were presented as mean ± SEM, unless otherwise indicated. Statistical analysis was performed using GraphPad Prism 8 software. An unpaired two-sided Student’s t-test was used to determine the p value, and p values were considered statistically significant below 0.05 (*p≤0.05; **p≤0.01; ***p≤0.001; ****p≤0.0001).

## Results

### Generation and characterization of a pan-allele anti-human SIRPα Antibody

Antibodies against human SIRPα were generated by immunization of mouse with SIRPα ECD. One of the positive clones (7C2) was obtained. 7C2 was then humanized by CDR grafting onto human germline frameworks and was constructed with a human IgG1 backbone. The N297A amino acid substitution in the Fc region was introduced in order to abrogate Fc-FcγR binding. The humanized version of 7C2 was designated as BR105.

**Figure 1:**
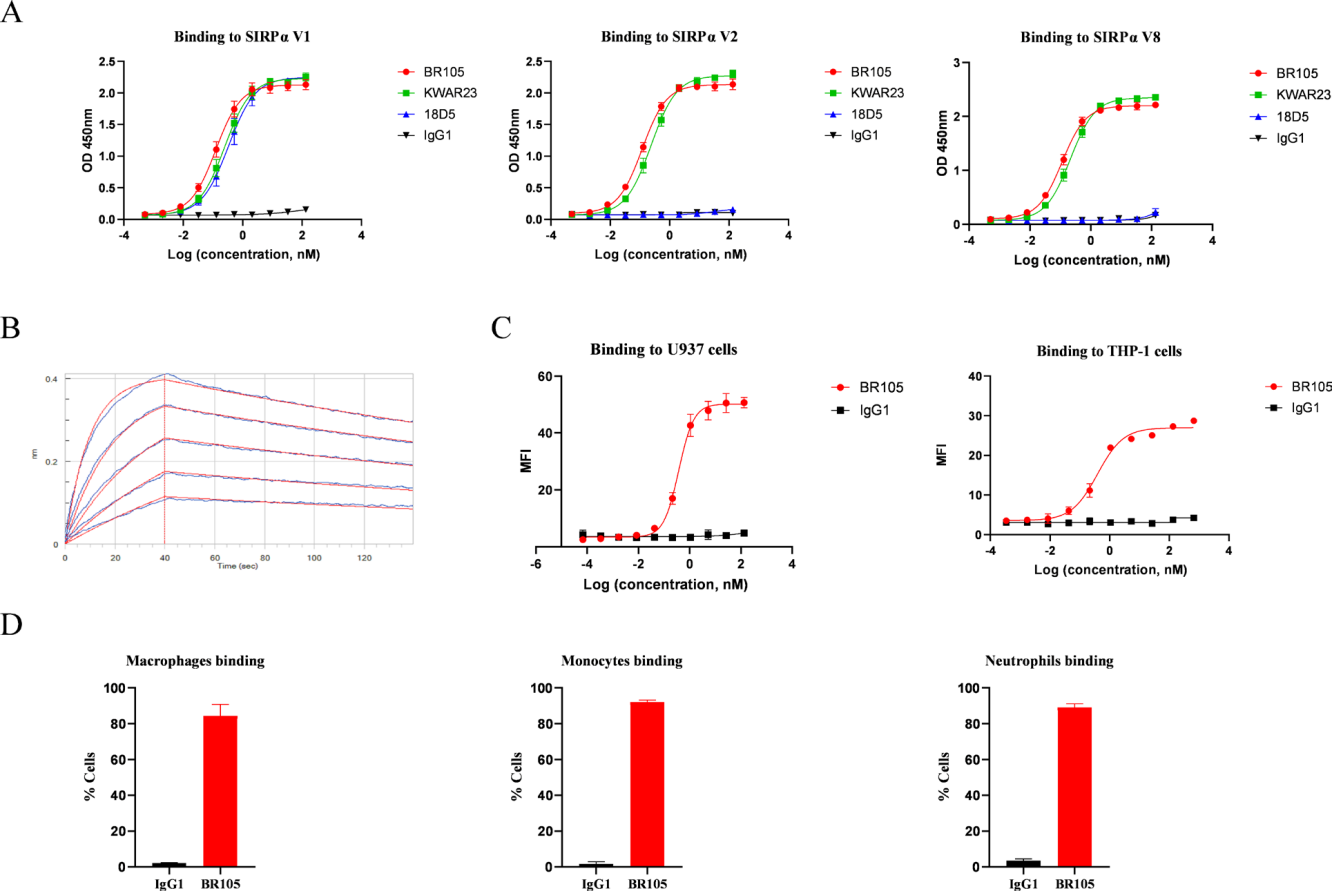
BR105 shows pan-allele anti-hSIRPα binding. (A) Binding of BR105 to various human SIRPα variant (V1, V2 and V8) was determined by ELISA. (B) Bio-Layer Interferometry analysis of the binding kinetics of BR105 to SIRPα V1. (C) Binding of BR105 to cell-expressed SIRPα V1 (U937) and SIRPα V2 (THP-1) was determined by flow cytometry analysis. (D) BR105 bound to monocytes, macrophages and neutrophils. Data represent mean ± SEM; representative of n=3 is shown.

The IgV domain of SIRPα is highly polymorphic and it has been reported that SIRPα V1, SIRPα V2, and SIRPα V8 are the main variants present in humans.^35 36^ A pan-allele anti-SIRPα antibody would be required for effective targeting of the SIRPα/CD47 checkpoint across diverse patient populations, as both alleles have to be inhibited to potentiate phagocytosis for heterozygous populations.^37^ The activity of BR105 binding to various SIRPα variants was tested by ELISA. Other SIRPα mAbs in development, including KWAR23 and 18D5, were used as controls. As shown in figure 1A, BR105 could recognize SIRPα V1, V2 and V8, displaying pan-allele SIRPα binding properties. Similarly, KWAR23 also bound to these SIRPα variants, as previously described.^36^ In contrast, 18D5 only bound to SIRPα V1. Bio-Layer Interferometry was used to analyze the binding kinetics of BR105. BR105 displayed high affinity, and bound to a monomeric human SIRPα with an affinity constant (KD) of 7.9 nM (figure 1B). FACS analysis revealed that BR105 bound to cell-expressed SIRPα V1 and SIRPα V2 on monocytic cell lines U937 and THP-1,^38^ respectively (figure 1C, online supplemental figure S1A, B). Moreover, BR105 was confirmed to bind to SIRPα expressed on human monocytes, macrophages and neutrophils (figure 1D, online supplemental figure S1C).

10.1136/jitc-2021-004054.supp1 Supplementary data

### BR105 shows potent antagonism of various SIRPα variants

**Figure 2:**
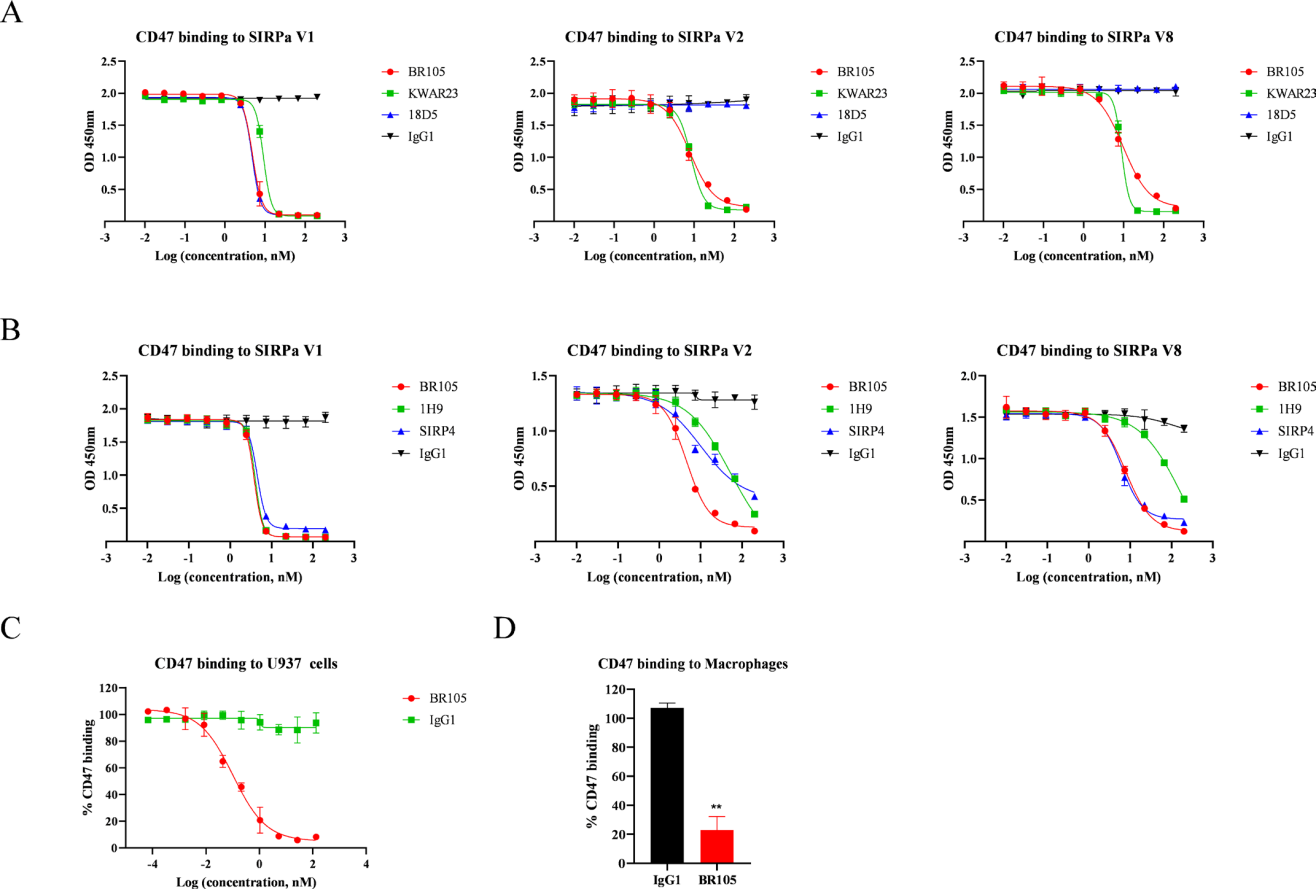
BR105 blocks CD47 binding to various SIRPα variants. (A, B) Blocking soluble CD47 binding to human SIRPα variants (V1, V2 and V8) was assessed by competition ELISA assay. Inhibiting of CD47 binding to SIRPα-expressing U937 (C) or macrophages (D) by BR105 was determined by flow cytometry analysis. Data represent mean ± SEM; representative of n=3 is shown. *indicate statistical differences compared with the respective isotype control group: **p<0.01.

We then assessed the ability of BR105 to block human CD47 from binding to SIRPα. In an ELISA competition assay, BR105 blocked the interaction between CD47 and SIRPα in a dose-dependent manner (figure 2A). BR105 showed comparable blocking activity toward the three main SIRPα alleles SIRPα V1, SIRPα V2 and SIRPα V8, indicating that BR105 has broad blocking activity across various SIRPα variants. In contrast, KWAR23 also blocked binding of CD47 to the three SIRPα variants, while 18D5 could only antagonize SIRPα V1. BR105 exhibited similar SIRPα blocking activity as KWAR23. However, compared with other pan-allele SIRPα mAbs in development, including 1H9 and SIRP4, BR105 showed better blocking activity against various SIRPα variants (figure 2B). We further evaluated the ability of BR105 to block CD47 binding to cell-expressed SIRPα. U937 cells were incubated with CD47-Fc fusion proteins either in the absence or presence of increasing concentrations of BR105. FACS analysis revealed potent antagonism of BR105 to cell-expressed SIRPα on U937 cells (figure 2C, online supplemental figure S2). In addition, BR105 also blocked the binding of SIRPα-expressing macrophages to human CD47 (figure 2D).

Because BR105, KWAR23, 1H9, 18D5 and SIRP4 could block the interaction between CD47 and SIRPα V1, we performed studies to assess whether these SIRPα mAbs bind to distinct or overlapping regions on SIRPα V1. Competitive ELISA assays suggest that BR105 does not compete with KWAR23 but compete with 18D5 for SIRPα binding (online supplemental figure S3). 18D5 and SIRP4 partially reduce the binding of BR105 to SIRPα, while 1H9 completely inhibits the binding of BR105. Similar to BR105, KWAR23 and 1H9 completely inhibit the binding of 18D5.

**Figure 3:**
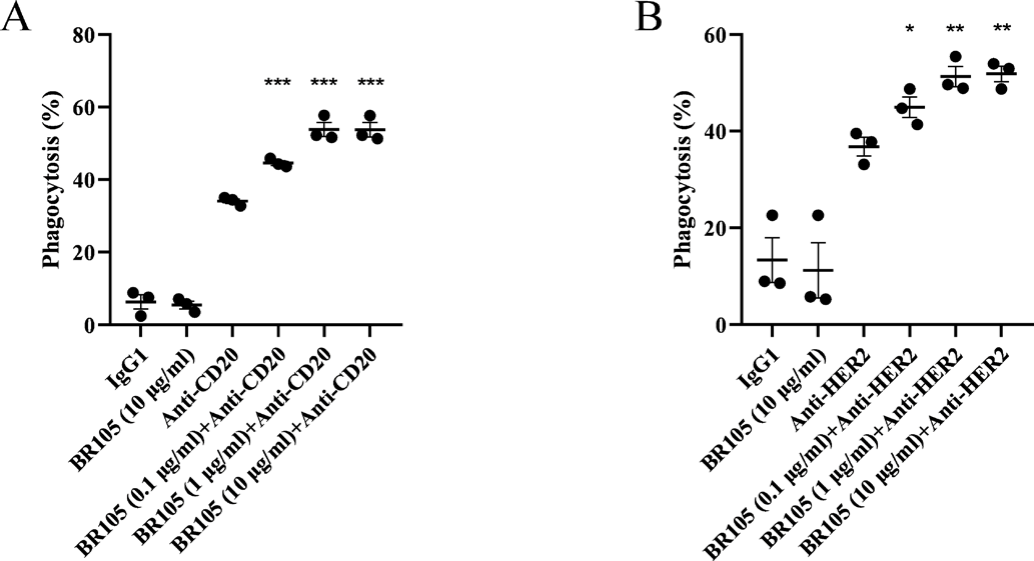
BR105 augments phagocytosis of tumor cells by macrophages. (A) Raji (human B-cell lymphoma line) and (B) SK-BR-3 (human breast cancer cell line) were incubated with human peripheral blood-derived macrophages (n=3 donors) in the presence of indicated concentrations of BR105, 0.5 µg/mL of anti-CD20, 0.5 µg/mL of anti-HER2, either alone or in combination. Phagocytosis was determined by flow cytometer. Data represent mean ± SEM; representative of n=3 donor is shown. *indicate statistical differences compared with the anti-CD20 (A) or anti-HER2 (B) group: *p<0.05, **p<0.01, ***p<0.001.

### BR105 enhances phagocytosis activity mediated by macrophages

We next evaluated the functional activity of BR105 to induce the phagocytosis of cancer cells by human monocytes-derived macrophages. An in vitro macrophage-based phagocytosis assay was performed, in which CD20-expressing Burkitt’s lymphoma Raji cells were co-incubated with human macrophages. As shown in figure 3A, BR105 alone did not induce phagocytosis activity. However, in combination with anti-CD20 antibody (zuberitamab), which is now being evaluated in phase 3 clinical trials, BR105 significantly enhanced phagocytosis of Raji cells by human macrophages obtained from different human individuals, and the synergistic activity between BR105 and anti-CD20 was seen across a range of concentrations of BR105 (figure 3A, online supplemental figure S4A). Similarly, BR105 augmented macrophage phagocytosis of SK-BR-3 breast cancer cells in the presence of anti-HER2 antibody (figure 3B, online supplemental figure S4B), which was developed by BioRay pharmaceutical as a biosimilar to trastuzumab. These results suggest that BR105 could promote therapeutic antibodies-mediated cell killing by macrophages.

### Cross-reactivity of BR105 to SIRPγ and the effect on T cell function

**Figure 4:**
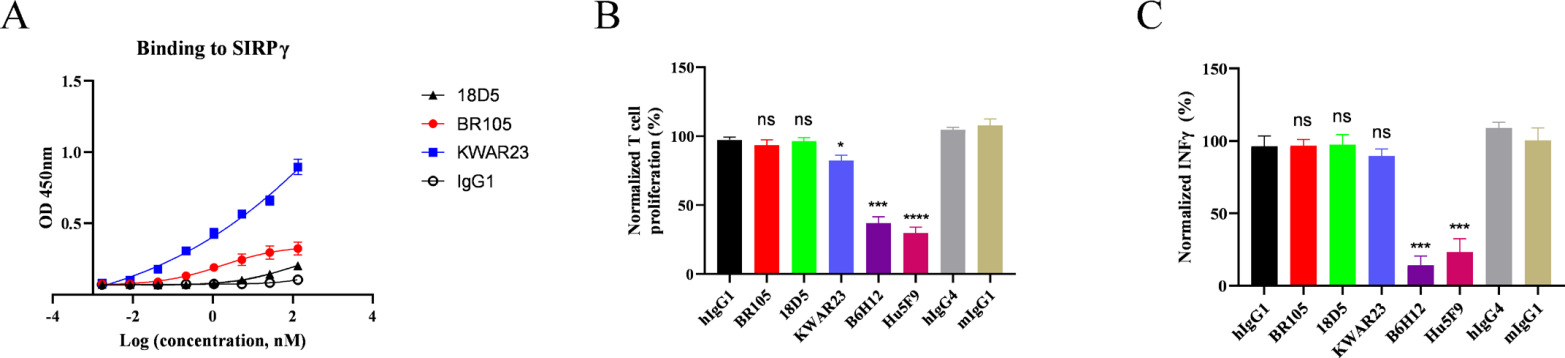
BR105 does not inhibit T cell activation and proliferation. (A) Binding of indicated mAbs to SIRPγ was determined by ELISA. Data represent mean±SEM; representative of n=3 is shown. (B) PBMCs were cultured with 10 µg/mL of indicated mAbs in OKT3-coated plates for 3 days. Proliferation measured by CellTiter-Glo was normalized to control (PBMCs without addition of mAbs). (C) Dendritic cells and allogeneic PBMCs from three different donors were cultured 5 days with indicated mAbs at 10 µg/mL. Interferon γ secretion was quantified by ELISA and normalized to control (Dendritic cells and PBMCs without addition of mAbs). Data represent mean ± SEM; representative of n=3 donor is shown. *Indicate statistical differences compared with the respective isotype control group: *p<0.05, ***p<0.001, ****p<0.0001. ns, not significant.

SIRPγ is a member of the SIRP family expressed on T cells and natural killer (NK) cells. It has been shown that the interaction of SIRPγ with CD47 on APCs was involved in T cell responses.^39^ We then evaluated the cross-reactivity of BR105 to SIRPγ and the effects of BR105 on T cell functionality. In the SIRPγ binding study, KWAR23, which was previously reported to bind to SIRPγ,^36^ was included as a control. As shown in figure 4A, KWAR23 could bind to SIRPγ. However, BR105 showed weak binding to SIRPγ, and the SIRPγ binding activity of BR105 was greatly reduced compared with that of KWAR23. We next investigated whether BR105 affected T cell activation in an OKT3-induced T-cell proliferation assay. No impairment of T cell proliferation was observed on stimulation with OKT3 in the presence of BR105, whereas KWAR23 and anti-CD47 mAbs, including B6H12 and Hu5F9, significantly inhibited T cell proliferation (figure 4B). Similarly, we found that anti-CD47 mAbs also reduced IFNγ secretion in an allogeneic mixed lymphocyte reaction (MLR) assay, while BR105 had no impact on IFNγ secretion (figure 4C). The above results suggest that although BR105 may weakly bind to SIRPγ, it does not inhibit activation and proliferation of T cells.

### In vivo antitumor activity of combination treatment with BR105

**Figure 5:**
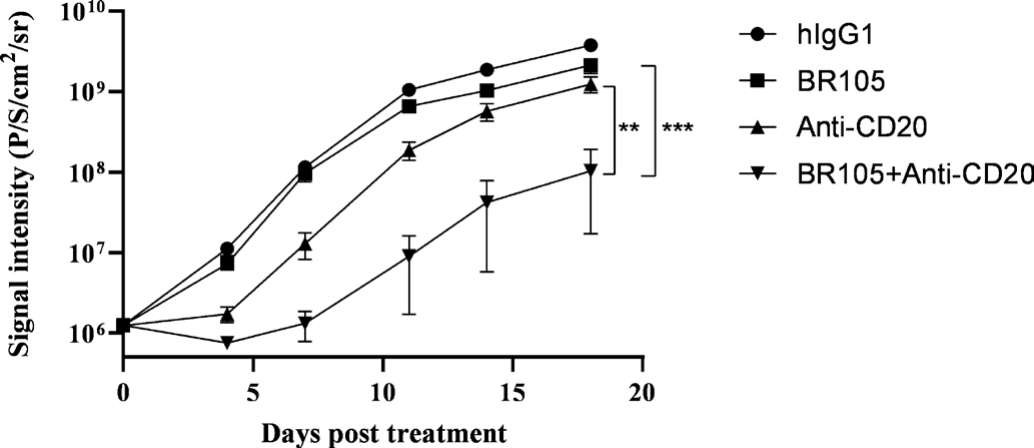
In vivo therapeutic activity of combination treatment with BR105. Raji-Luciferase cells were engrafted intravenously into hSIRPα B-NDG mice (n=8 for each group). 3 days post engraftment, treatment was initiated with human IgG1 (hIgG1), BR105, anti-CD20, or combination of BR105 and anti-CD20. Total flux measurements were obtained two times per week to assess tumor growth. Data represent mean ± SEM. *indicate statistical differences compared with the respective single agent control group: **p<0.01, ***p<0.001.

We evaluated in vivo efficacy of BR105 alone or in combination with anti-CD20 antibody (zuberitamab) by employing hSIRPα-humanized B-NDG mice. Mice transplanted intravenously with Raji-luciferase cells were assigned to treatment with either human IgG1 control, BR105, anti-CD20 (zuberitamab), or BR105 combined with anti-CD20, and then followed by in vivo bioluminescent imaging to determine tumor growth. Treatment with BR105 or anti-CD20 alone showed no or minimal effects on tumor growth, whereas the combination of BR105 and anti-CD20 led to significant inhibition of tumor growth compared with either agent treatment alone (figure 5, online supplemental figure S5). Our results suggest that BR105 synergizes with anti-CD20 to exert antitumor efficacy in vivo.

### Assessment of BR105 safety and pharmacokinetics

**Figure 6:**
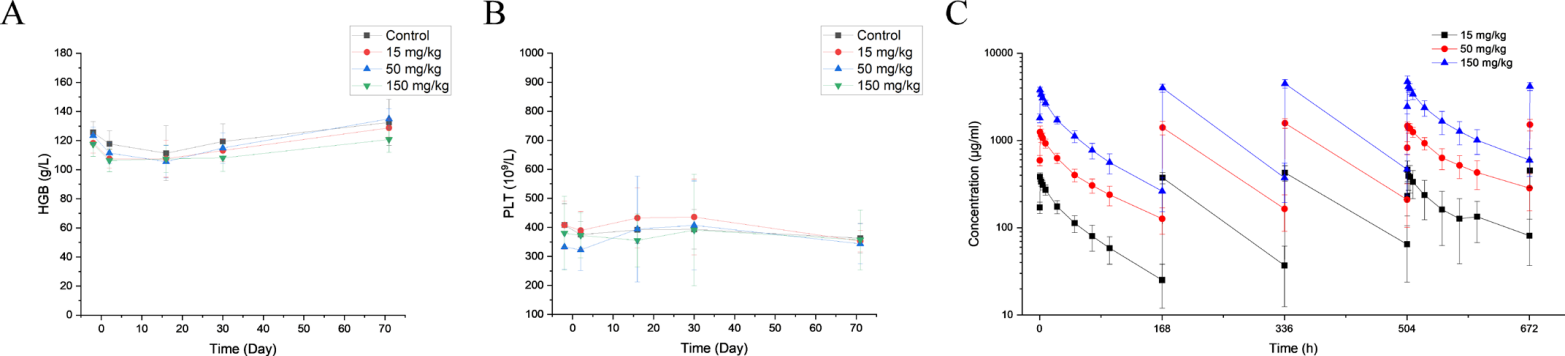
BR105 can be safely administered intravenously in non-human primates. BR105 was administered intravenously to cynomolgus monkeys at 15, 50, and 150 mg/kg (five males and five females for each group) one time per week for 4 weeks. Following the dosing period, two animals/sex per group were maintained for a 6-week recovery period. (A, B) Peripheral blood was collected for hematology during the pre-dose phase; on Days 2 and 16 of the dosing phase; and on Days 30 and 71 of the recovery period. (C) Serum concentration-time profiles of BR105 following intravenous repeat doses in cynomolgus monkeys (semi-logatithmic). Values are presented as mean ± SD.

The pharmacokinetic and toxicology profiles of BR105 were investigated in cynomolgus monkeys. Cynomolgus monkey SIRPα exhibits a high degree amino acid sequence identity (more than 90%) with corresponding human SIRPα. BR105 binds to cynomolgus monkey SIRPα with an EC50 of 0.016 nM (online supplemental figure S6), comparable to its binding activity for human SIRPα (figure 1). A 4-week repeat-dose GLP-compliant toxicology study with a 6-week recovery period was conducted in cynomolgus monkeys. The administration of BR105 was well tolerated at all dose levels. No abnormal clinical signs and BR105-related changes in body weight or food consumption were observed during the study (online supplemental figures S7, 8). There were no BR105-related changes in ophthalmologic examinations or cardiovascular safety pharmacology evaluations (online supplemental figure S9). There were also no BR105-related effects on hematology or clinical chemistry parameters (online supplemental figures S10-12). Transient anemia and thrombocytopenia were not observed after BR105 administration when compared with the control group (figure 6A, B). In addition, there were no BR105-related effects on gross pathology, relative organ weights, or histopathology (online supplemental table S1, figure S13). These findings show that BR105 may have a favorable safety profile.

Serum concentration of BR105 were measured and analyzed by PK modeling. Following four weekly intravenous administration of BR105, approximately dose-proportional increases in maximum concentration (Cmax) and area under the concentration-time curve (AUC) were seen across the dose range 15 to 150 mg/kg (figure 6C). Serum accumulation of BR105 was observed following intravenous infusion one time per week for 4 weeks. The accumulation ratios for AUC_last_ ranged from 1.3 to 1.7.

## Discussion

Myeloid cells, including macrophages, dendritic cells and neutrophils, are the most abundant cells in the tumor microenvironment (TME). The TME reprograms infiltrating myeloid cells into immunosuppressive tumor-associated macrophages (TAMs) or myeloid-derived suppressor cells (MDSCs) to sustain an immunosuppressive environment. The tumor-infiltrating myeloid cells stimulate tumor angiogenesis, suppress tumor immunity, promote metastasis, and are associated with immune checkpoint therapy resistance and poor prognosis.^40^ Targeting TAM and MDSCs may represent a promising approach to control tumor progression and prevent metastasis of cancer cells. SIRPα is established as a myeloid inhibitory immunoreceptor, which can interact with CD47 on cancer cells and send ‘don't eat me’ signal to phagocytes. The CD47/SIRPα axis has emerged as one of the most promising cancer immunotherapy targets. Various CD47/SIRPα blocking agents have entered clinical trials. Some CD47-targeting agents, including Hu5F9, ALX148 and TTI-621, have shown encouraging clinical efficacy in hematological malignancies, especially when using in combination with other agents.^19 22 24^ It has been reported that CD47-targeting agents can cause anemia or thrombocytopenia.^22–24^ The on-target anemia observed with Hu5F9 was mitigated by the strategy of prime and maintenance dosing.^16^ Other Anti-CD47 antibodies with negligible red blood cell binding properties have been developed and are currently being tested in clinical trials.^41^ The long-term safety and durability of clinical responses in patients treated with these CD47-targeting agents are needed to be further evaluated. In addition, the widespread CD47 expression in many normal tissues is thought to create an ‘antigen sink’, which may affect the bioavailability and receptor occupancy of CD47-targeting agents. Given its more restricted histological distribution, direct targeting of SIRPα is anticipated to overcome these challenges and provide an alternative strategy for cancer immunotherapy.

Here, we described a novel humanized anti-SIRPα antibody BR105. BR105 binds human SIRPα with high affinity and blocks the interaction between CD47 and SIRPα. BR105 promotes phagocytosis of antibody-opsonized tumor cells and synergizes with therapeutic antibodies to inhibit tumor growth in vivo. SIRPα polymorphism presents challenges for the development of effective SIRPα blocking antibodies. SIRPα variants with polymorphism in the amino-terminal ligand binding domain have been reported across diverse human populations. Among the SIRPα variants, SIRPα V1, SIRPα V2, and SIRPα V8 are the most predominant variants, constituting approximately 90% of SIRPα present in human population.^35 36^ The SIRPα V1 allele is mainly found in European, Admixed American, South Asian and African populations, while SIRPα V2 is the most prominent allele among the East Asian population. The SIRPα polymorphism was thought to be the result of evolutionary pressure following binding of pathogens or pathogen products to the inhibitory receptor.^42^ BR105 could bind to SIRPα V1, SIRPα V2 and SIRPα V8, and showed potent antagonism of these SIRPα variants. As a pan-allele–specific anti-SIRPα antibody, BR105 is anticipated to have clinical efficacy in a wide range of patients.

SIRPγ is another member of the SIRP family and is expressed in T cells and NK cells. The extracellular domain of SIRPγ is highly homologous to SIRPα, and no known signaling motifs are found in the cytoplasmic domain of SIRPγ, suggesting a lack of intrinsic signaling capacity. SIRPγ could also bind to CD47 but with a 10-fold lower affinity than SIRPα.^43^ It has been shown that the interaction between CD47 and SIRPγ is involved in cell‐to‐cell adhesion, T cell activation and cytokine secretion.^39^ Some antibodies against CD47 or SIRPγ resulted in inhibition of T cell proliferation.^36 39 44^ BR105 showed a very slight binding to SIRPγ. We further tested whether BR105 impaired T cell function. We found that BR105 did not inhibit OKT3-induced T-cell proliferation, and did not change IFNγ levels in allogeneic MLR assay. These indicated that BR105 had no impact on T cell function. In contrast, treatment with anti-CD47 agents or anti-SIRPα antibody KWAR23 inhibited T cell activation.

Our data suggest that SIRPα targeting by BR105 enhances macrophage-mediated phagocytosis of tumor cells when used in combination with the anti-CD20 antibody (zuberitamab) developed in our laboratory, which is now being evaluated in phase 3 clinical trials, and has shown encouraging efficacy in diffuse large B-cell lymphoma. Moreover, BR105 synergizes with anti-CD20 to inhibit tumor growth in a B-NDG mice model, although BR105 alone has no obvious effect. Phagocytosis is tightly regulated by prophagocytic (‘eat me’) and anti-phagocytic (‘don’t eat me’) signals. Engagement of activating FcRs on immune cells induces protein tyrosine phosphorylation and provides a robust stimulus for macrophage activation. It has been shown that SIRPα protein or anti-CD47 antibody without an Fc portion was unable to induce phagocytosis,^21^ implying the disruption of the SIRPα–CD47 interaction may not be enough to induce robust antitumor immunity. Combined with tumor-opsonizing antibodies that retain FcγR binding capacity, SIRPα-blocking agents can effectively induce phagocytosis and display enhanced antitumor responses.^32 36 45^ These also provides strategy for anti-CD47 antibodies to achieve tumor selectivity, thereby reducing on-target off-tumor toxicity toward healthy CD47-expressing cells. In addition, other receptor on macrophages, including LRP-1, SLAMF7 and Mac-1, may also be involved in prophagocytic signals during the SIRPα–CD47 blockade.^46 47^ However, evidence suggests that intrinsical activating signals are frequently insufficient to support single agent SIRPα-CD47 blockade.^21 32 37^ Further clarifying activating signal and addition inhibitory signals presented on myeloid cells may help to improve our ability to predict the outcome of SIRPα-CD47 inhibition.

We showed that BR105 could bind and activate macrophages, leading to enhanced phagocytosis of cancer cells. Studies in syngeneic mouse models suggest that anti-SIRPα antibody modifies the tumor microenvironment with an enhancement in the M1/M2 macrophage ratio.^44 48^ Tumor-associated macrophages can be divided into M1 and M2 phenotypes, which are thought to have anti-tumorigenic and pro-tumorigenic activities, respectively. The increased M1/M2 macrophage ratio may favor promoting the antitumor immune response. Besides the innate immune system, the adaptive immune system may also contribute to the tumor growth inhibition on the SIRPα-CD47 pathway blockade. In an ovalbumin (OVA) model antigen system, OVA-specific CD8+ T cells exhibited increased cytotoxicity toward OVA-positive tumor cells on injection of macrophages that had been co-cultured with OVA-expressing target cells in the presence of anti-CD47 antibody.^49^ The study suggests that the SIRPα–CD47 blockade augments the ability of macrophages to stimulate antigen-specific CD8+ T cells responses. In another study, cross-priming of CD8+ T cells by dendritic cell, rather than dendritic cell, was thought to be critical for the therapeutic antitumor effect of anti-CD47 treatment.^50^ It seems feasible that enhanced antigen-presentation on the SIRPα–CD47 blockade contributes to CD8+ T cell responses. Moreover, it has been reported that SIRPα inhibition induced macrophage chemokine secretion, stimulated tumor nest T cell recruitment and increased antitumor T cell responses.^44^ Collectively, the above studies suggest that targeting the SIRPα–CD47 immune checkpoint could bridge innate and adaptive immunity and may induce a more durable immune response. The exact mechanism by which the CD47-SIRPα blockade regulates T cell function remains to be elucidated.

In summary, we developed a novel humanized anti-SIRPα antibody BR105 and performed preclinical assessment of BR105. BR105 binds with high affinity to human SIRPα and blocks binding of SIRPα to CD47. Moreover, BR105 displays broad activity across various SIRPα variants. We further showed that BR105 synergizes with therapeutic antibodies to potentiate phagocytosis of tumor cells in vitro and to inhibit tumor growth in vivo. Furthermore, toxicology studies in NHP suggest BR105 displays a favorable safety profile. These findings support further development of BR105 in clinical combination strategies for antitumor therapy.

## Conclusion

BR105 is a differentiating anti-SIRPα antibody that binds to major SIRPα variants and activate macrophages to lead enhanced phagocytosis of tumor cells. BR105 shows favorable safety profiles over other non-selective SIRPα-targeting antibodies and CD47-targeting agents. Thus, BR105 is a promising agent to be further developed for cancer immunotherapy.

## Data Availability

Data are available in a public, open access repository. Data are available upon reasonable request. All data relevant to the study are included in the article or uploaded as supplementary information.

## References

[1] Ni L, Dong C. New checkpoints in cancer immunotherapy. Immunol Rev 2017;276:52–65. 10.1111/imr.12524 28258699

[2] Hodi FS, O'Day SJ, McDermott DF, et al. Improved survival with ipilimumab in patients with metastatic melanoma. N Engl J Med 2010;363:711–23. 10.1056/NEJMoa1003466 20525992 PMC3549297

[3] McDermott DF, Drake CG, Sznol M, et al. Survival, durable response, and long-term safety in patients with previously treated advanced renal cell carcinoma receiving nivolumab. J Clin Oncol 2015;33:2013–20. 10.1200/JCO.2014.58.1041 25800770 PMC4517051

[4] Robert C, Ribas A, Hamid O, et al. Durable complete response after discontinuation of pembrolizumab in patients with metastatic melanoma. J Clin Oncol 2018;36:1668–74. 10.1200/JCO.2017.75.6270 29283791

[5] Reck M, Rodríguez-Abreu D, Robinson AG, et al. Pembrolizumab versus chemotherapy for PD-L1-positive non-small-cell lung cancer. N Engl J Med 2016;375:1823–33. 10.1056/NEJMoa1606774 27718847

[6] Hamid O, Robert C, Daud A, et al. Five-year survival outcomes for patients with advanced melanoma treated with pembrolizumab in KEYNOTE-001. Ann Oncol 2019;30:582–8. 10.1093/annonc/mdz011 30715153 PMC6503622

[7] Restifo NP, Smyth MJ, Snyder A. Acquired resistance to immunotherapy and future challenges. Nat Rev Cancer 2016;16:121–6. 10.1038/nrc.2016.2 26822578 PMC6330026

[8] Sharma P, Hu-Lieskovan S, Wargo JA, et al. Primary, adaptive, and acquired resistance to cancer immunotherapy. Cell 2017;168:707–23. 10.1016/j.cell.2017.01.017 28187290 PMC5391692

[9] Weiskopf K. Cancer immunotherapy targeting the CD47/SIRPalpha axis. Eur J Cancer 2017;76:100–9. 10.1016/j.ejca.2017.02.013 28286286

[10] Veillette A, Chen J. SIRPalpha-CD47 immune checkpoint blockade in anticancer therapy. Trends Immunol 2018;39:173–84. 10.1016/j.it.2017.12.005 29336991

[11] Matlung HL, Szilagyi K, Barclay NA, et al. The CD47-SIRPalpha signaling axis as an innate immune checkpoint in cancer. Immunol Rev 2017;276:145–64. 10.1111/imr.12527 28258703

[12] Oldenborg PA, Zheleznyak A, Fang YF, et al. Role of CD47 as a marker of self on red blood cells. Science 2000;288:2051–4. 10.1126/science.288.5473.2051 10856220

[13] Oldenborg P-A. Role of CD47 in erythroid cells and in autoimmunity. Leuk Lymphoma 2004;45:1319–27. 10.1080/1042819042000201989 15359629

[14] Majeti R, Chao MP, Alizadeh AA, et al. CD47 is an adverse prognostic factor and therapeutic antibody target on human acute myeloid leukemia stem cells. Cell 2009;138:286–99. 10.1016/j.cell.2009.05.045 19632179 PMC2726837

[15] Willingham SB, Volkmer J-P, Gentles AJ, et al. The CD47-signal regulatory protein alpha (SIRPa) interaction is a therapeutic target for human solid tumors. Proc Natl Acad Sci U S A 2012;109:6662–7. 10.1073/pnas.1121623109 22451913 PMC3340046

[16] Liu J, Wang L, Zhao F, et al. Pre-clinical development of a humanized Anti-CD47 antibody with anti-cancer therapeutic potential. PLoS One 2015;10:e0137345. 10.1371/journal.pone.0137345 26390038 PMC4577081

[17] Andrejeva G, Capoccia BJ, Hiebsch RR, et al. Novel SIRPalpha antibodies that induce single-agent phagocytosis of tumor cells while preserving T cells. J Immunol 2021;206:712–21. 10.4049/jimmunol.2001019 33431660 PMC7851740

[18] Chao MP, Alizadeh AA, Tang C, et al. Anti-CD47 antibody synergizes with rituximab to promote phagocytosis and eradicate non-Hodgkin lymphoma. Cell 2010;142:699–713. 10.1016/j.cell.2010.07.044 20813259 PMC2943345

[19] Kauder SE, Kuo TC, Harrabi O, et al. ALX148 blocks CD47 and enhances innate and adaptive antitumor immunity with a favorable safety profile. PLoS One 2018;13:e0201832. 10.1371/journal.pone.0201832 30133535 PMC6104973

[20] Sockolosky JT, Dougan M, Ingram JR, et al. Durable antitumor responses to CD47 blockade require adaptive immune stimulation. Proc Natl Acad Sci U S A 2016;113:E2646–54. 10.1073/pnas.1604268113 27091975 PMC4868409

[21] Weiskopf K, Ring AM, Ho CCM, et al. Engineered SIRPα variants as immunotherapeutic adjuvants to anticancer antibodies. Science 2013;341:88–91. 10.1126/science.1238856 23722425 PMC3810306

[22] Advani R, Flinn I, Popplewell L, et al. CD47 blockade by Hu5F9-G4 and rituximab in non-Hodgkin’s lymphoma. N Engl J Med 2018;379:1711–21. 10.1056/NEJMoa1807315 30380386 PMC8058634

[23] Sikic BI, Lakhani N, Patnaik A, et al. First-in-human, first-in-class phase I trial of the anti-CD47 antibody Hu5F9-G4 in patients with advanced cancers. J Clin Oncol 2019;37:946–53. 10.1200/JCO.18.02018 30811285 PMC7186585

[24] Ansell SM, Maris MB, Lesokhin AM, et al. Phase I study of the CD47 blocker TTI-621 in patients with relapsed or refractory hematologic malignancies. Clin Cancer Res 2021;27:2190–9. 10.1158/1078-0432.CCR-20-3706 33451977

[25] Soto-Pantoja DR, Kaur S, Roberts DD. CD47 signaling pathways controlling cellular differentiation and responses to stress. Crit Rev Biochem Mol Biol 2015;50:212–30. 10.3109/10409238.2015.1014024 25708195 PMC4822708

[26] Gao L, Chen K, Gao Q, et al. CD47 deficiency in tumor stroma promotes tumor progression by enhancing angiogenesis. Oncotarget 2017;8:22406–13. 10.18632/oncotarget.9899 27283989 PMC5410232

[27] Adams S, van der Laan LJ, Vernon-Wilson E, et al. Signal-regulatory protein is selectively expressed by myeloid and neuronal cells. J Immunol 1998;161:1853–9. 9712053

[28] Veillette A, Thibaudeau E, Latour S. High expression of inhibitory receptor SHPS-1 and its association with protein-tyrosine phosphatase SHP-1 in macrophages. J Biol Chem 1998;273:22719–28. 10.1074/jbc.273.35.22719 9712903

[29] Oldenborg PA, Gresham HD, Lindberg FP. CD47-signal regulatory protein alpha (SIRPalpha) regulates Fcgamma and complement receptor-mediated phagocytosis. J Exp Med 2001;193:855–62. 10.1084/jem.193.7.855 11283158 PMC2193364

[30] Tsai RK, Discher DE. Inhibition of "self" engulfment through deactivation of myosin-II at the phagocytic synapse between human cells. J Cell Biol 2008;180:989–1003. 10.1083/jcb.200708043 18332220 PMC2265407

[31] Poirier N, Mary C, Vanhove B. New anti-SIRPa antibodies and their therapeutic applications. Geneva, Switzerland: World Intellectual Property Organization, 2017.

[32] Ring NG, Herndler-Brandstetter D, Weiskopf K, et al. Anti-SIRPα antibody immunotherapy enhances neutrophil and macrophage antitumor activity. Proc Natl Acad Sci U S A 2017;114:E10578–85. 10.1073/pnas.1710877114 29158380 PMC5724266

[33] Liu J, Volkmer JP. Anti-sirp-alpha antibodies and related methods. Geneva, Switzerland: World Intellectual Property Organization, 2018.

[34] Puro R, Manning PT, Karr RW. Therapeutic SIRPα antibodies. Geneva, Switzerland: World Intellectual Property Organization, 2019.

[35] Takenaka K, Prasolava TK, Wang JCY, et al. Polymorphism in SIRPa modulates engraftment of human hematopoietic stem cells. Nat Immunol 2007;8:1313–23. 10.1038/ni1527 17982459

[36] Voets E, Paradé M, Lutje Hulsik D, et al. Functional characterization of the selective pan-allele anti-SIRPα antibody ADU-1805 that blocks the SIRPα-CD47 innate immune checkpoint. J Immunother Cancer 2019;7:340. 10.1186/s40425-019-0772-0 31801627 PMC6894304

[37] Zhao XW, van Beek EM, Schornagel K, et al. CD47-signal regulatory protein-α (SIRPα) interactions form a barrier for antibody-mediated tumor cell destruction. Proc Natl Acad Sci U S A 2011;108:18342–7. 10.1073/pnas.1106550108 22042861 PMC3215076

[38] Tsai RK, Rodriguez PL, Discher DE. Self inhibition of phagocytosis: the affinity of 'marker of self' CD47 for SIRPalpha dictates potency of inhibition but only at low expression levels. Blood Cells Mol Dis 2010;45:67–74. 10.1016/j.bcmd.2010.02.016 20299253 PMC2878922

[39] Piccio L, Vermi W, Boles KS, et al. Adhesion of human T cells to antigen-presenting cells through SIRPbeta2-CD47 interaction costimulates T-cell proliferation. Blood 2005;105:2421–7. 10.1182/blood-2004-07-2823 15383453

[40] Ugel S, De Sanctis F, Mandruzzato S, et al. Tumor-induced myeloid deviation: when myeloid-derived suppressor cells meet tumor-associated macrophages. J Clin Invest 2015;125:3365–76. 10.1172/JCI80006 26325033 PMC4588310

[41] Puro RJ, Bouchlaka MN, Hiebsch RR, et al. Development of AO-176, a next-generation humanized Anti-CD47 antibody with novel anticancer properties and negligible red blood cell binding. Mol Cancer Ther 2020;19:835–46. 10.1158/1535-7163.MCT-19-1079 31879362

[42] Hatherley D, Lea SM, Johnson S, et al. Polymorphisms in the human inhibitory signal-regulatory protein α do not affect binding to its ligand CD47. J Biol Chem 2014;289:10024–8. 10.1074/jbc.M114.550558 24550402 PMC3974974

[43] Brooke G, Holbrook JD, Brown MH, et al. Human lymphocytes interact directly with CD47 through a novel member of the signal regulatory protein (SIRP) family. J Immunol 2004;173:2562–70. 10.4049/jimmunol.173.4.2562 15294972

[44] Gauttier V, Pengam S, Durand J, et al. Selective SIRPα blockade reverses tumor T cell exclusion and overcomes cancer immunotherapy resistance. J Clin Invest 2020;130:6109–23. 10.1172/JCI135528 33074246 PMC7598080

[45] Ho CCM, Guo N, Sockolosky JT, et al. "Velcro" engineering of high affinity CD47 ectodomain as signal regulatory protein α (SIRPα) antagonists that enhance antibody-dependent cellular phagocytosis. J Biol Chem 2015;290:12650–63. 10.1074/jbc.M115.648220 25837251 PMC4432284

[46] Chen J, Zhong M-C, Guo H, et al. SLAMF7 is critical for phagocytosis of haematopoietic tumour cells via Mac-1 integrin. Nature 2017;544:493–7. 10.1038/nature22076 28424516 PMC5565268

[47] Chao MP, Jaiswal S, Weissman-Tsukamoto R, et al. Calreticulin is the dominant pro-phagocytic signal on multiple human cancers and is counterbalanced by CD47. Sci Transl Med 2010;2:63ra94. 10.1126/scitranslmed.3001375 PMC4126904 21178137

[48] Yanagita T, Murata Y, Tanaka D, et al. Anti-SIRP**α** antibodies as a potential new tool for cancer immunotherapy. JCI Insight 2017;2:e89140. 10.1172/jci.insight.89140 28097229 PMC5214103

[49] Tseng D, Volkmer J-P, Willingham SB, et al. Anti-CD47 antibody-mediated phagocytosis of cancer by macrophages primes an effective antitumor T-cell response. Proc Natl Acad Sci U S A 2013;110:11103–8. 10.1073/pnas.1305569110 23690610 PMC3703977

[50] Liu X, Pu Y, Cron K, et al. CD47 blockade triggers T cell-mediated destruction of immunogenic tumors. Nat Med 2015;21:1209–15. 10.1038/nm.3931 26322579 PMC4598283

