# Supplementary material for: Preclinical characterization of the novel anti-SIRPα antibody BR105 that targets the myeloid immune checkpoint
Source: J Immunother Cancer. 2022 Mar 7;10(3):e004054. doi: 10.1136/jitc-2021-004054 (PMC8905892; doi:10.1136/jitc-2021-004054)
Supplement: Supplementary data [file jitc-2021-004054supp001.pdf]

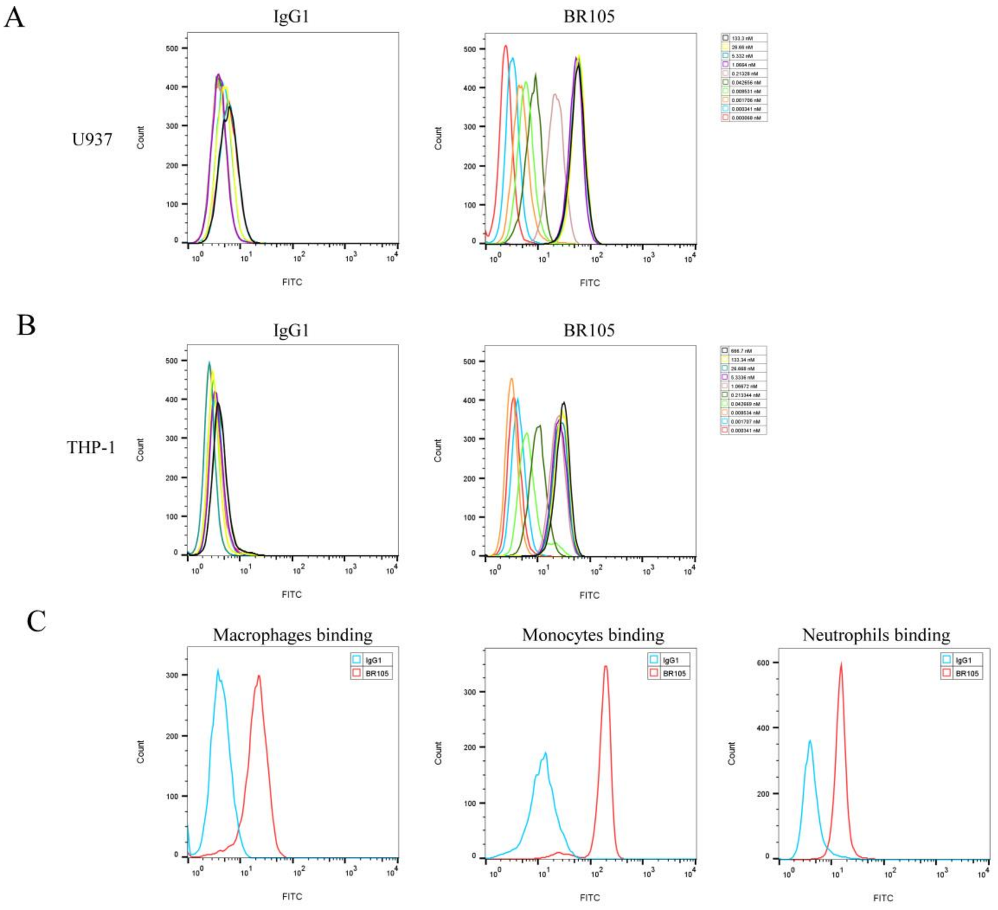

Figure. S1 Representative flow cytometry plots of isotype control IgG1 and BR105 binding to U937 (A), THP-1 (B) and primary cells (C).

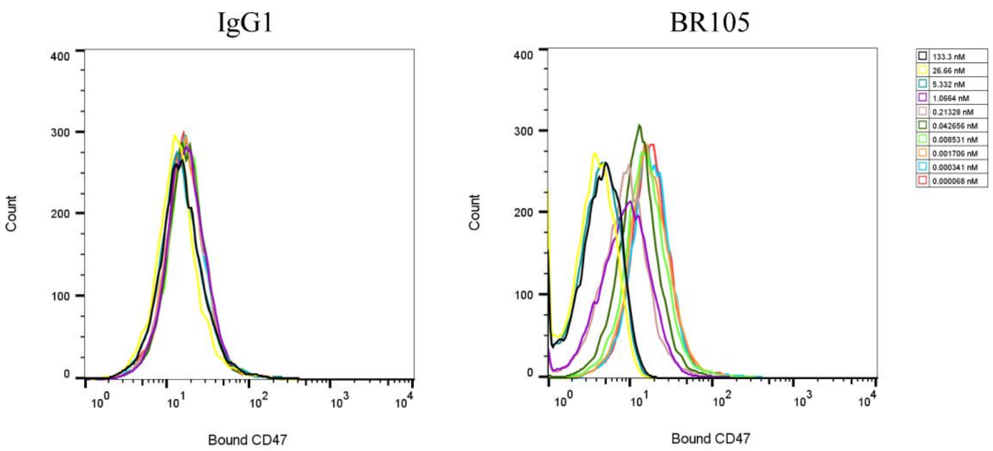

Figure. S2 Sample data to determine inhibition of soluble CD47 binding by BR105 to SIRPα-expressing cells, data for U937 cell line shown.

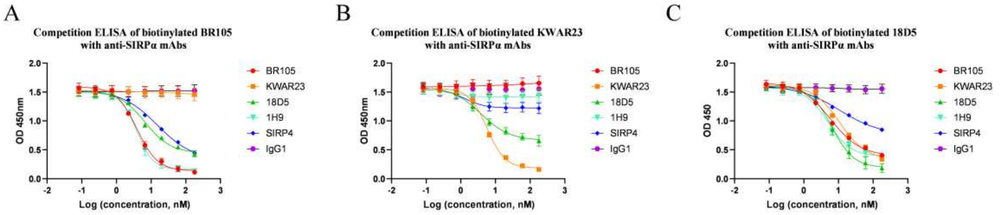

Figure. S3 Epitope competitive ELISA assays for binding of BR105, KWAR23, 1H9, 18D5 and SIRP4 to human SIRPα V1.

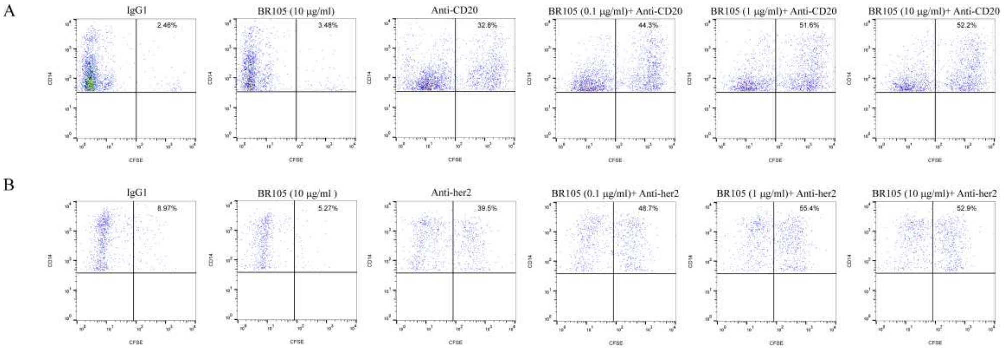

Figure. S4 Representative dot plots of CD14+ macrophages co-cultured with CFSE-labeled Raji (A) or SK-BR-3 (B) to determine the percentage of macrophages that have engulfed tumor cells.

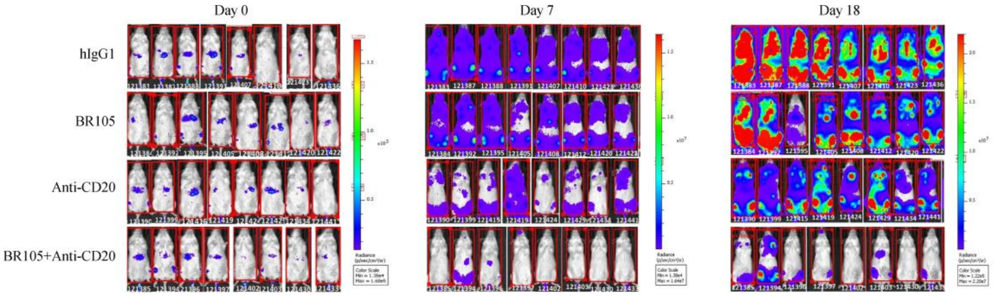

Figure. S5 Bioluminescence images of Raji xenograft mice performed on the indicated days after initiation of treatment with human IgG1, BR105, anti-CD20 (zuberitamab), or combination of BR105 and anti-CD20 (zuberitamab).

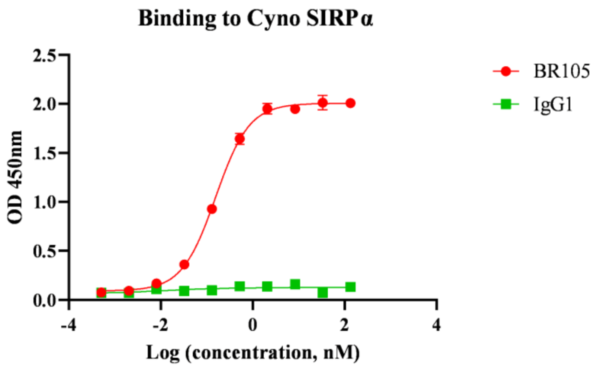

Figure. S6 BR105 binding to cynomolgus monkey SIRPα was assessed by ELISA.

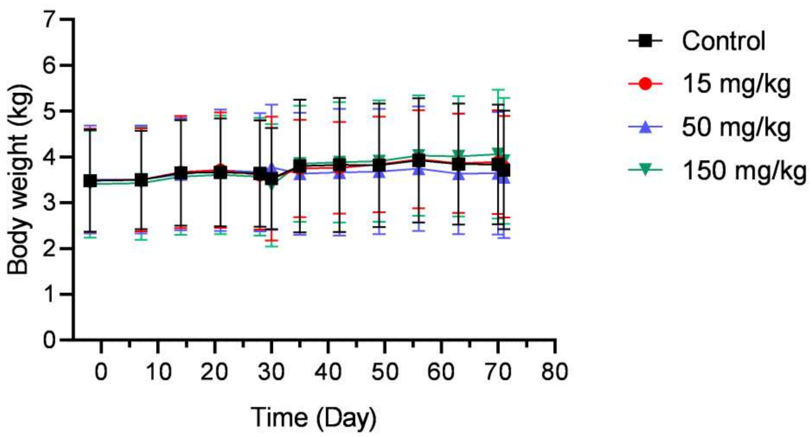

Figure. S7 Effects of BR105 on body weight in cynomolgus monkeys. Values are presented as mean ± SD.

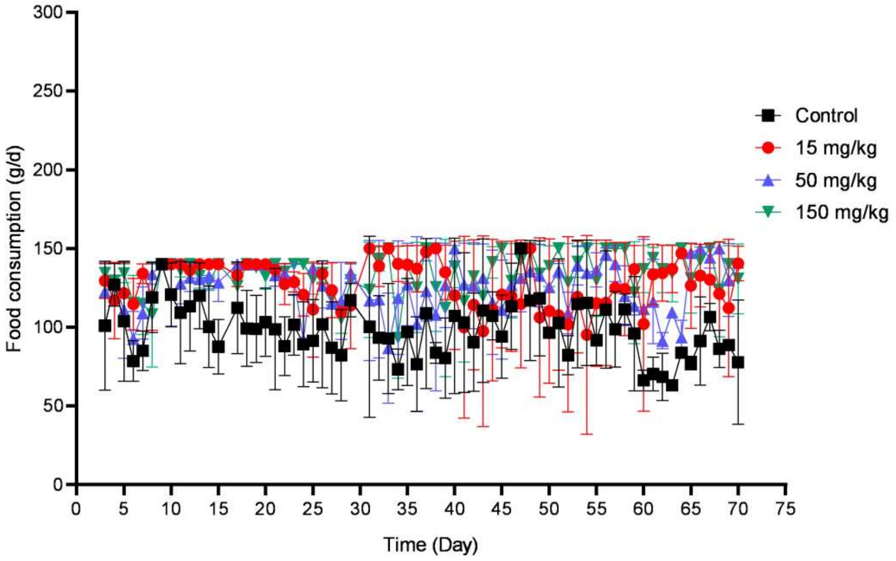

Figure. S8 Effects of BR105 on food consumption in cynomolgus monkeys. Values are presented as mean ± SD.

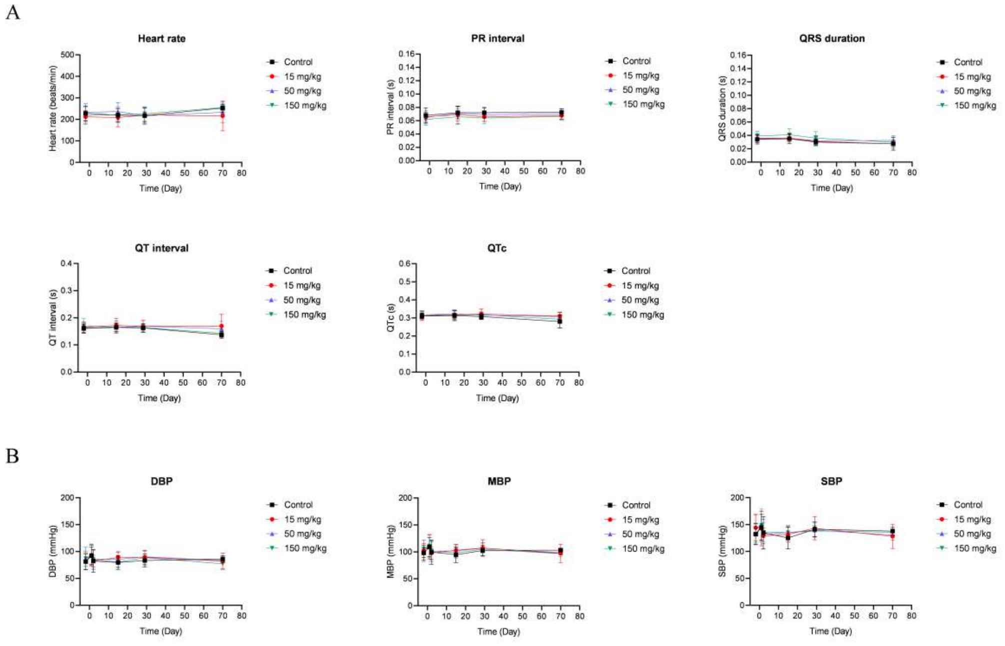

Figure. S9 Electrocardiogram evaluation (A) and blood pressure measurement (B) in cynomolgus monkeys following intravenous administration of BR105. Values are presented as mean ± SD.

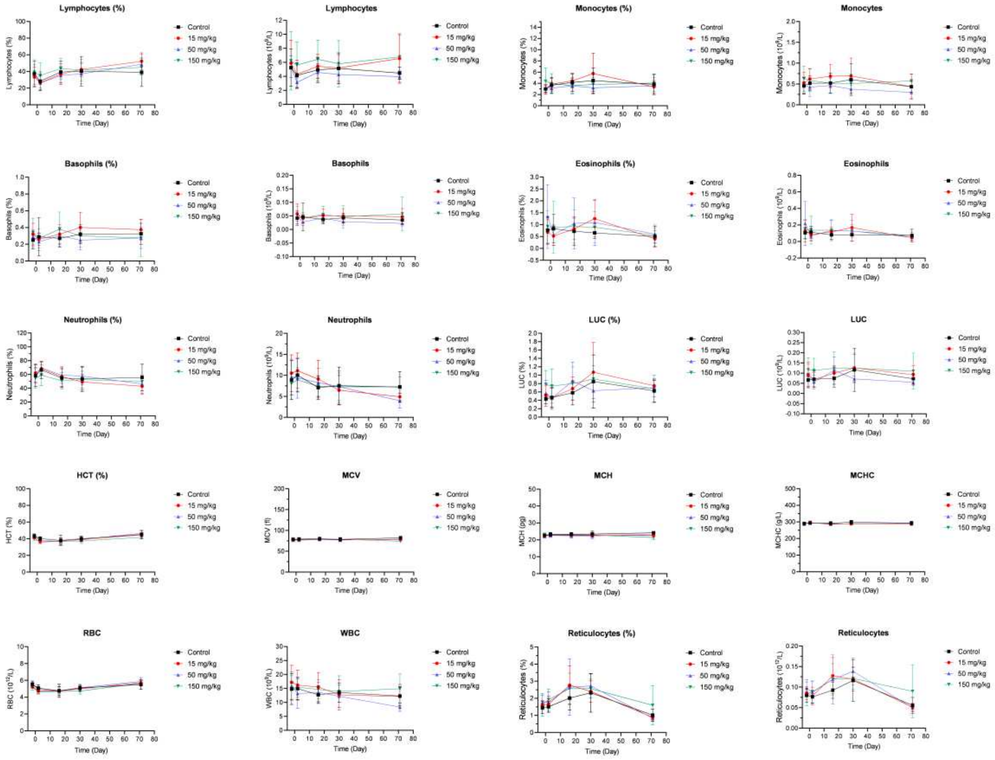

Figure. S10 Hematology parameters in cynomolgus monkeys following intravenous administration of BR105. Values are presented as mean ± SD.

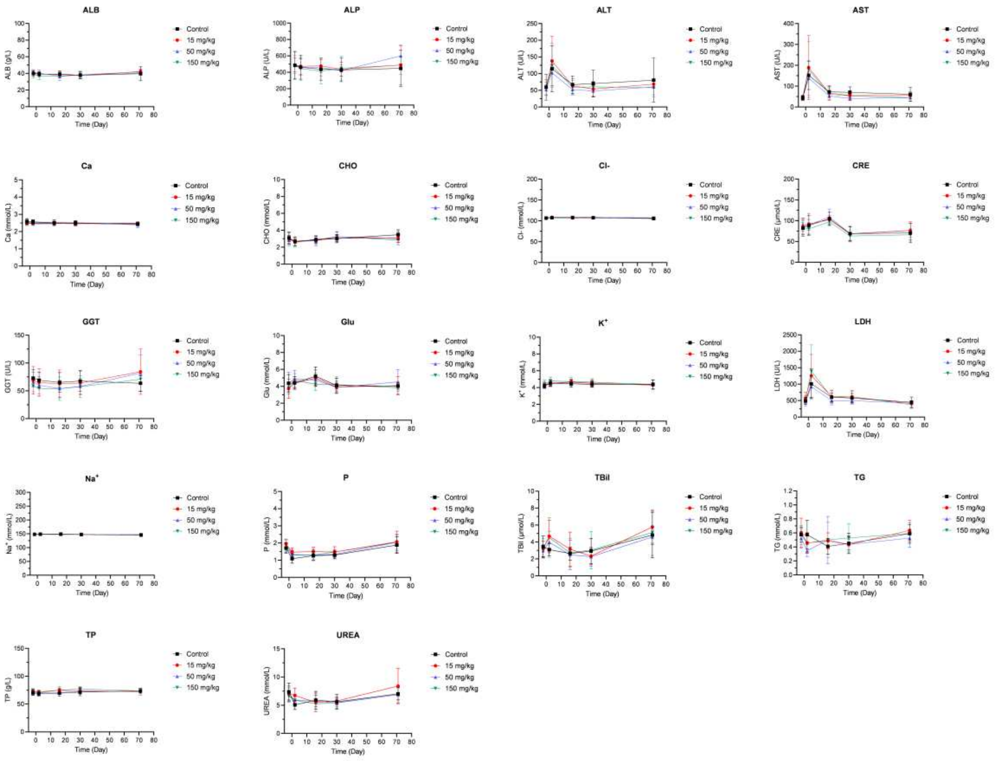

Figure. S11 Serum chemistry measurements in cynomolgus monkeys following intravenous administration of BR105. Values are presented as mean ± SD.

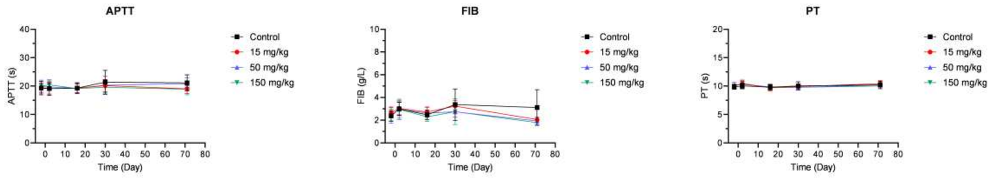

Figure. S12 Coagulation parameters evaluation following intravenous administration of BR105 in cynomolgus monkeys. Values are presented as mean ± SD.

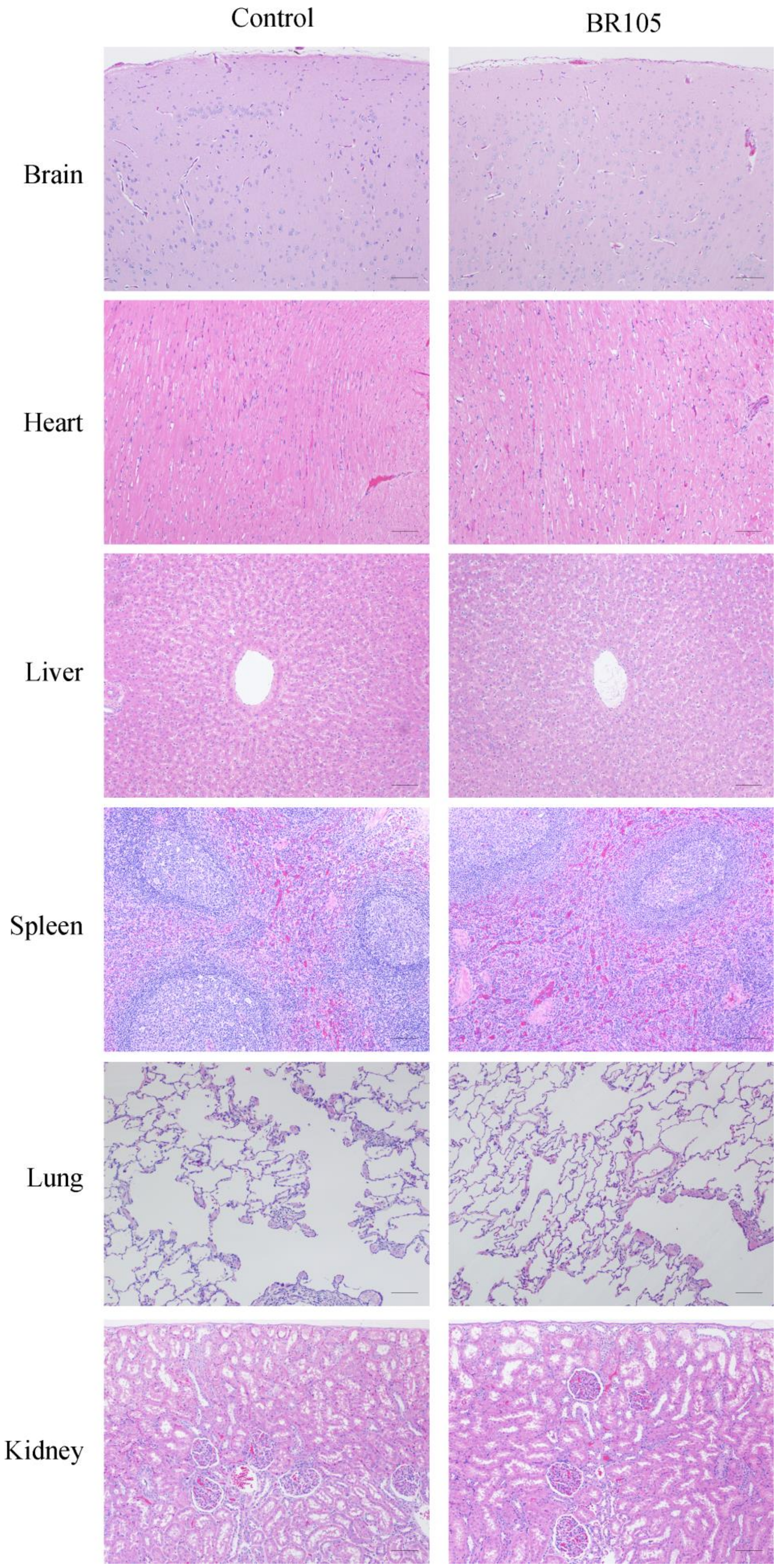
Control
BR105
Brain
Heart
Liver
Spleen
Lung
Kidney

Figure. S13 Representative photomicrographs of HE staining in brain, heart, liver, spleen, lung and kidney tissue from cynomolgus monkeys following intravenous administration of BR105 (150 mg/kg). Scale bars = 100 μm.

Table S1 Effect of administering BR105 on relative organ weight (%) of cynomolgus monkeys

Organ	Drug treatment			
	0 mg/kg	15 mg/kg	50 mg/kg	150 mg/kg
Brain	2.138±0.615	2.096±0.713	1.909±0.600	2.210±0.741
Thymus	0.037±0.023	0.045±0.025	0.045±0.040	0.064±0.035
Heart	0.343±0.047	0.391±0.070	0.367±0.032	0.378±0.038
Lung and main bronchi	0.494±0.062	0.493±0.052	0.454±0.043	0.528±0.064
Liver	1.764±0.270	1.943±0.249	1.854±0.295	1.944±0.282
Spleen	0.130±0.059	0.139±0.079	0.157±0.061	0.183±0.115
Kidney	0.350±0.063	0.386±0.102	0.393±0.126	0.387±0.076
Adrenal gland	0.015±0.005	0.018±0.006	0.016±0.005	0.017±0.005
Thyroid and parathyroid glands	0.009±0.002	0.012±0.003	0.009±0.003	0.011±0.005
Pituitary	0.001±0.001	0.002±0.001	0.002±0.001	0.002±0.001

All values are Mean ± SD (n=6).
